# Enhancing ultrafiltration performance for dairy wastewater treatment using a 3D printed turbulence promoter

**DOI:** 10.1007/s11356-023-30027-4

**Published:** 2023-09-27

**Authors:** Aws N. Al-Tayawi, Nikolett Sz. Gulyás, Gréta Gergely, Ákos Ferenc Fazekas, Balázs Szegedi, Cecilia Hodúr, József Richárd Lennert, Szabolcs Kertész

**Affiliations:** 1 Doctoral School of Environmental Sciences, University of Szeged, Szeged, H-6725 Hungary; 2 Department of Environmental Technology, Faculty of Environmental Science and Technology, University of Mosul, Mosul, 41002 Iraq; 3 Department of Food Engineering, Faculty of Engineering, University of Szeged, Szeged, H-6725 Hungary; 4 Department of Biosystems Engineering, Faculty of Engineering, University of Szeged, Szeged, H-6725 Hungary; 5 Department of Mechanical Engineering, Faculty of Engineering, University of Szeged, Szeged, H-6725 Hungary; 6 Department of Power Electronics and E-Drives, Audi Hungaria Faculty of Automotive Engineering, Széchenyi István University, Győr, H-9026 Hungary

**Keywords:** 3DP turbulence promoter, Dairy wastewater treatment, Membrane fouling mitigation, Ultrafiltration, Stirring speed

## Abstract

Dairy factories annually generate an increasing amount of wastewater, which can cause eutrophication due to high concentrations of amino acids and lipids. To address this issue, membrane technology has emerged as a promising solution, but membrane fouling remains a significant challenge, since it can cause decreased flux, decrease membrane rejection performance, and increased energy demand. This study aimed to reduce membrane fouling by integrated a three-dimensional printed (3DP) turbulence promoter into an ultrafiltration dead-end cell and varying stirring speeds. Two mathematical models, Hermia and resistance-in-series, were used to analyze the fouling process. According to both models, the cake layer formation model indicated the most prevalent fouling mechanism. Specific energy demand, permeate flux, membrane rejection, and membrane reversible and irreversible resistances were measured, calculated, and compared. The results suggest that the combination of an integrated 3DP turbulence promoter and high stirring speeds can effectively reduce membrane fouling in a dairy wastewater treatment module.

## Introduction

In recent years, there has been a significant increase in the amount of dairy wastewater discharged annually due to the growing demand for dairy products (Ji et al. 2020; Miao et al. 2021). The dairy industry is one of the world’s staple industries and is considered the largest industrial food wastewater source, particularly in Europe (Kolev Slavov 2017). However, the high levels of proteins and lipids in dairy wastewater pose challenges for treatment processes, as they alter the pH value and increase the organic content (Mohebrad et al. 2022). The resultant pollution from the leakage of untreated dairy wastewater into the environment is a growing concern (Deka et al. 2022). Conventional technologies such as distillation, adsorption, and extraction are insufficient to meet the disposal limits for industrial wastewater treatment (Basumatary et al. 2015). Therefore, biological and physicochemical treatment technologies are typically used to treat dairy wastewater. Although physicochemical approaches have been shown to be effective in removing chemical oxygen demand (COD), they are costly due to the use of chemical coagulants (Kumar et al. 2016) In recent years, membrane technology has emerged as a promising alternative for the treatment of dairy wastewater due to its efficiency in reducing organic compounds (Gong et al. 2012; Catenacci et al. 2020). Several membrane separation technologies, including reverse osmosis (RO), nanofiltration (NF), ultrafiltration (UF), and microfiltration (MF), have been used to treat dairy wastewater (Ahmad and Ahmed 2014; Gonsalves et al. 2023).

However, the current challenges and limitations of membrane filtration processes are membrane fouling and concentration polarization (Tan et al. 2014; Leu et al. 2017). Membrane fouling is a significant issue, as it can decrease flux, decrease rejection performance, and increase energy consumption (Saffarimiandoab et al. 2021). Fouling occurs through adhesion and deposition of foulants and filtration of foulant layers, which can be particulate, colloidal particles or matter, biomacromolecules, and organic, inorganic, and biological substances in various forms (Ferreira et al. 2020). Nonspecific adhesion of microorganisms and biomacromolecules to the membrane surface results in blocked or significantly reduced membrane pores, leading to decreased permeation flux or separation efficiency (Ladewig and Al-Shaeli 2017).

To address this issue, researchers are exploring the use of three-dimensional printed (3DP) turbulence promoters integrated into membrane modules (Ju et al. 2022). These 3DP elements offer innovative opportunities to mitigate fouling by optimizing membrane modules with turbulence promoters (Armbruster et al. 2018; Soo et al. 2021). This study aims to investigate the integration of a 3DP turbulence promoter into an ultrafiltration cell and to evaluate its performance at different stirring speeds. Two mathematical models, the Hermia and resistance-in-series model, will be applied to identify the prevailing fouling mechanism.

The main objectives of the study are to assess the impact of 3DP turbulence promoters on the mitigation of membrane fouling and to analyze changes in hydrodynamic conditions and shear rate on the membrane surface. The experiments will measure the specific energy demand, permeate flux, and membrane rejection to evaluate the effectiveness of the integrated 3DP turbulence promoter. This study builds on a previous experiment (Kertész et al. 2023), where various 3DP turbulence promoters were tested and the most promising one was chosen for this study. By integrating 3DP turbulence promoters into dairy wastewater treatment membrane modules, the research aims to develop more efficient and sustainable solutions for dairy wastewater treatment and water desalination.

## Materials and methods

### Preparation of the dairy wastewater model

A wastewater model was prepared by dissolution of 50 g of skimmed milk powder (Tutti Kft., Hungary) and 5 g of anionic detergent Cl80 (Sole-Mizo Zrt., Szeged, Hungary) in 10 L of tap water at a controlled temperature of 25 °C, resulting in a final concentration of 5 g·L^−1^. The homogenization process was achieved using a magnetic stirrer for a duration of 30 min. The chosen concentration of 5 g·L^−1^ was based on the typical organic load observed in wastewater generated by the dairy industry, as reported in the literature (Posavac et al. 2010; Shete, Bharati, and Shete and Shinkar 2013; Kolev Slavov 2017).

### Construction of membrane separation equipment

Laboratory membrane separation experiments were conducted using a Millipore Solvent Resistant Stirred Micro- and Ultrafiltration Cell (Merck Millipore in Darmstadt, Germany). The cell is composed of a stainless steel bottom and a borosilicate glass wall. It has an active membrane surface area of 40 cm^2^, enabling rapid concentration of laboratory volume samples of up to 300 mL at a maximum pressure of 5.5 bar.

For this study, a 150 kDa molecular weight cutoff point (MWCO) membrane was used, which had a pore size of 0.07 μm, a thickness of 250 μm, and a hydrophilicity characterized by an average contact angle of 60°. This indicates that the membrane is hydrophilic (Sawada et al. 2012). In a previous study (Kertész et al. 2014), the hydrophilicity of a polyethersulfone (PES) membrane with a MWCO of 10 kDa was investigated. The contact angle of the PES membrane was found to decrease from 43 to 37.7° with a rate of 12.3% over a period of 240 s. Additionally, Nasrollahi et al. (2018) reported an average water contact angle value of 70.2° for the bare PES UF membrane. They compared this result with their own-produced blended PES membranes containing different concentrations of the CuO/ZnO nanocomposite.

The membrane was placed at the bottom of the ultrafiltration cell on a thin support mesh. The transmembrane pressure (TMP) required for filtration was applied using nitrogen gas (Messer, Hungary). The system was continuously stirred using a magnetic stirrer suspended in the middle of the cell. The permeate flux was collected through a plastic tube at the bottom of the cell, and the amount of permeate was measured using a scale at the outlet within the volume reduction ratio (VRR) of 2.

### Application and characteristics of the 3DP turbulence promoter

The experiment used the most optimal design of a single 3DP turbulence promoter, which was previously selected based on our prior research (Kertész et al. 2023). These promoters were fabricated using PLA (polylactic acid) material, chosen for its superior properties compared to ABS (acrylonitrile butadiene styrene) (Atakok et al. 2022; San Andrés et al. 2023). The fabrication process involved fused deposition modeling (FDM) technology, using a Creality CR-10S Pro V2 3D printer (Shenzhen, China). The printing parameters included a layer thickness of 0.2 mm, a 100% fill density, a tray temperature of 60 °C, and a printing temperature of 215 °C. The design of the promoters was carried out using Fusion 360 Autodesk software (San Francisco, CA, USA), while slicing was performed using the Ultimaker Cura 5.0.0 program (Utrecht, The Netherlands).

The turbulence promoter had specific dimensions: an outer layer with a diameter of 65 mm, an inner layer with a smaller diameter of 39 mm, and a height of 14 mm, comprising 18 panels. The promoter was placed on the membrane’s surface using two circular rings in its bottom half. The outer ring was securely attached to the sealing O-ring and remained stable even during stirring. The promoter’s frame was constructed by interconnecting baffles between the circular rings, and the number and arrangement of these baffles had a significant impact on the flow conditions of the separated materials.

### Typical indicators of membrane operations

The performance or efficiency of a specific membrane can be characterized by specifying several parameters. One of the most important is permeability, or flux, the volume of filtrate passing through the membrane per unit of time, and unit of area. This can be calculated based on Eq. (1) below (Shen et al. 2022):

1 $$J=\frac{dV_P}{dt}\bullet \frac{1}{A_M}\ \left[\textrm{L}\bullet {\textrm{m}}^{-2}\bullet {\textrm{h}}^{-1}\right]$$

where *J* is the permeate flux [L∙m^−2^∙h^−1^], *V*_*P*_ is the volume of the permeate [L], *A*_M_ is the active surface of the membrane [m^2^], and *t* is the time [h].

The membrane’s selectivity can be characterized by the retention, which indicates the percentage of the initial solution remaining in the retentate for the given component (e.g., *COD*). Retention can be described by the following Eq. (2) (Lahnafi et al. 2022):

2 $$R=\left(1-\frac{c_P}{c_F}\right)\bullet 100\ \left[\%\right]$$

where *R* is retention [%], *c*_*P*_ is the concentration of the solution in the permeate [mg∙L^−1^ for *COD*], and *c*_*F*_ is the concentration of the solution on the feed side (Lahnafi et al. 2022).

### Modeling

#### Resistance-in-series model

During membrane separation operations, the efficiency of the process deteriorates over time, leading to a decline in the flux values. This decline can be attributed to concentration polarization or membrane fouling, which can be determined by monitoring changes in resistance values. Ideally, the resistance value should only reflect the membrane’s resistance (*R*_*M*_) (Anis et al. 2022). The hydrodynamic resistance of the pristine membrane can be calculated by measuring the water flux prior to filtration, as this eliminates the formation of a polarization layer on the membrane surface and prevents pore fouling (Dippel et al. 2021). The resistance of the pure membrane can be derived using Eq. (3) (Xu and Chen 2021).

3 $${R}_M=\frac{TMP}{J_{WB}\bullet {\eta}_W}\ [m^{-1}]$$

where *TMP* is the transmembrane pressure, thus the main driving force of membrane separation [Pa], *J*_*WB*_ is the water flux before filtration [L∙m^−2^∙h^−1^], and *ηw* is the dynamic viscosity of the water at 25 °C [Pa.s].

**Fig. 1:**
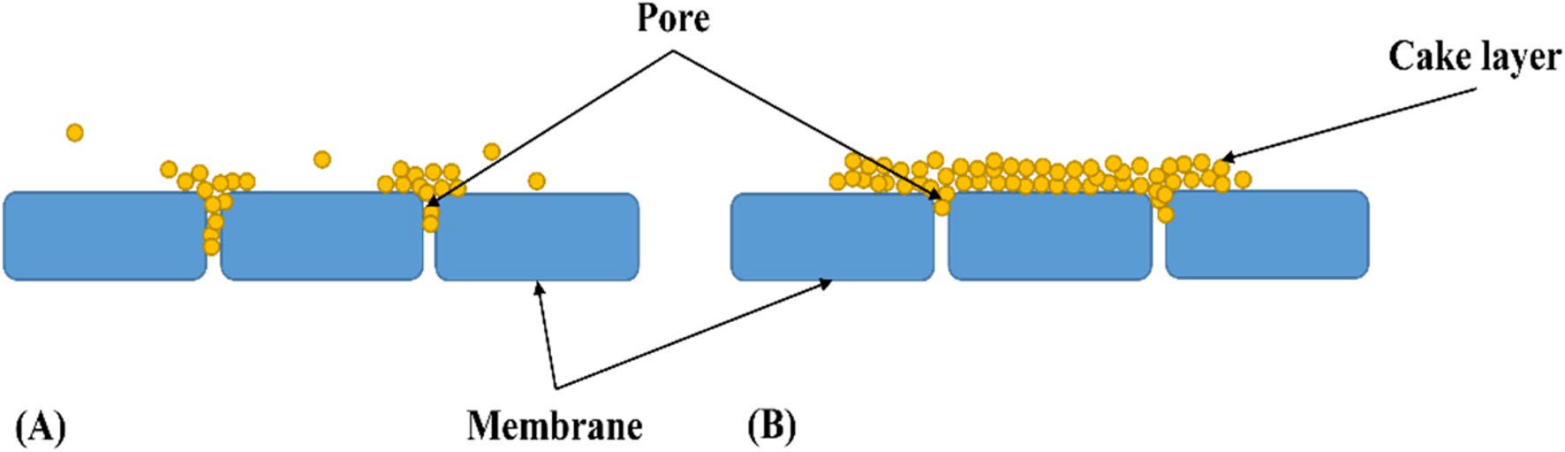
Principle of the development of (**A**) irreversible resistance and (**B**) reversible resistance

Quantification of resistances caused by pore fouling, namely, irreversible resistance (*R*_*IRREV*_) and reversible resistance (*R*_*REV*_), requires the dismantling of the module and subsequent rinsing of the membrane surface. The irreversible resistance can be obtained through Eq. (4) as proposed by Xu and Chen (2021). Furthermore, Fig. 1(A) shows a graphical representation of irreversible resistance.

4 $${R}_{IRREV}=\left(\frac{TMP}{J_{WA}\bullet {\eta}_W}-{R}_M\right)\ [m^{-1}]$$

where *J*_*WA*_ is the water flux of the membrane [L∙m^−2^∙h^−1^] after filtration and *R*_*M*_ is the resistance of the membrane [%].

The value of the reversible resistance can be determined by the following Eq. (5) (Xu and Chen 2021). The principle of reversible resistance is shown in Fig. 1(B):

5 $${R}_{REV}=\left(\frac{TMP}{J_{WW}\times {\eta}_{WW}}\right)- R_{M}-{R}_{IRREV}\kern0.5em [m^{-1}]$$

where *J*_*WW*_ is the constant flux measured during wastewater filtration [L∙m^−2^∙ h^−1^], and *η*_*WW*_ is the dynamic viscosity of wastewater at 25 °C [Pa.s].

In practice, the total resistance (*R*_*T*_), Eq. (6), consists of three resistances: membrane resistance, reversible resistance, and irreversible resistance (Szerencsés et al. 2021).

6 $${R}_T={R}_M+{R}_{IRREV}+{R}_{REV}\kern0.5em [m^{-1}]$$

#### Hermia model

The Hermia model is a useful tool for characterizing and interpreting the effects of 3DP turbulence promoters and membrane fouling in membrane filtration processes. Semi-empirical and empirical mathematical models, such as the Hermia module, are commonly employed to analyze these phenomena. The Hermia model, initially developed by Hermia in 1982, provides a semi-empirical mathematical approach to describe the decline in permeate flux. This model is based on traditional constant pressure filtration methods and has been widely used to characterize membrane occlusion. The models of complete blocking, intermediate blocking, standard blocking, and cake layer have been defined using the Hermia model (Sreedhar et al. 2022).

#### Reynolds number

The hydrodynamic conditions in a membrane filtration cell were investigated by calculating the Reynolds number (*N*_*Re*_), a dimensionless parameter in fluid mechanics that characterizes fluid flow patterns by relating inertial and viscous forces. At low Reynolds numbers, laminar flow dominates, whereas at high Reynolds numbers, turbulence arises due to fluctuations in fluid speed and direction, resulting in eddy currents that consume energy and promote cavitation in liquids. The Reynolds number plays a vital role in predicting the onset of turbulence and scaling effects, which can aid in forecasting fluid behavior on a larger scale. The Reynolds number (*N*_*Re*_) can be expressed as Eq. (7) (Rehm et al. 2008):

7 $${N}_{Re}=\frac{\rho \upsilon d}{\mu }$$

where *ρ* is the density of the solution [kg·m^–3^], *υ* is the velocity [m·s^−1^], *d* is the diameter of the magnetic stirrer [m], and *μ* is the viscosity of the solution [Pa.s].

## Results and discussion

### Permeate fluxes

**Fig. 2:**
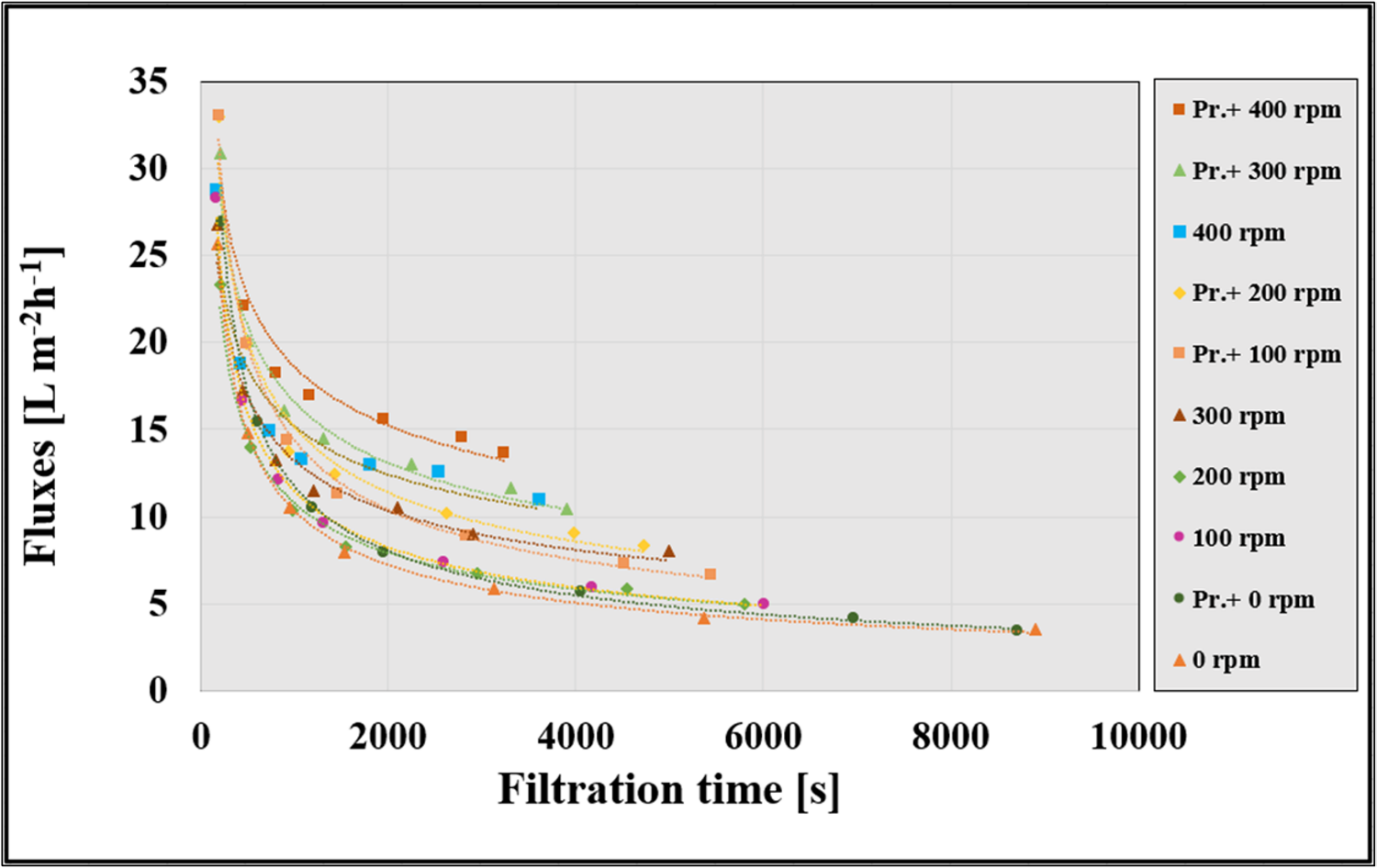
Changes in permeate fluxes as a function of time during ultrafiltration

Following laboratory measurements, the permeate fluxes were calculated and compared with Eq. (1) to determine the filtration speed. Figure 2 shows the flows during ultrafiltration at different stirring speeds with and without the insertion of the 3DP turbulence promoter into the cell (indicated by a robust trendline). After the initial intense downward trend as a result of rapid surface deposition, a settling phase follows. In particular, the integration of the 3DP turbulence promoter led to higher average fluxes in all cases. The total ultrafiltration times were measured to VRR of 2. The slowest time was observed in the control condition, 0 rpm, at 8900 s, while the fastest was in the condition mixed with the 3DP turbulence promoter, at 400 rpm, resulting in 3240 s, a 2.7-time difference. These findings underscore the importance of the integration of the 3DP turbulence promoter in ultrafiltration processes. Armbruster et al. (2018) indicated that static mixers that feature varying diameters are shown to be less efficient compared to twisted tape mixers with a consistent diameter, resulting in an approximately 130% increase in permeate flux. The most significant improvement in flux, reaching 140%, is observed when a Kenics mixer is employed. Irrespective of their geometries, all examined static mixers lead to higher permeate fluxes at the same specific energy consumption.

### Hermia model results

**Fig. 3:**
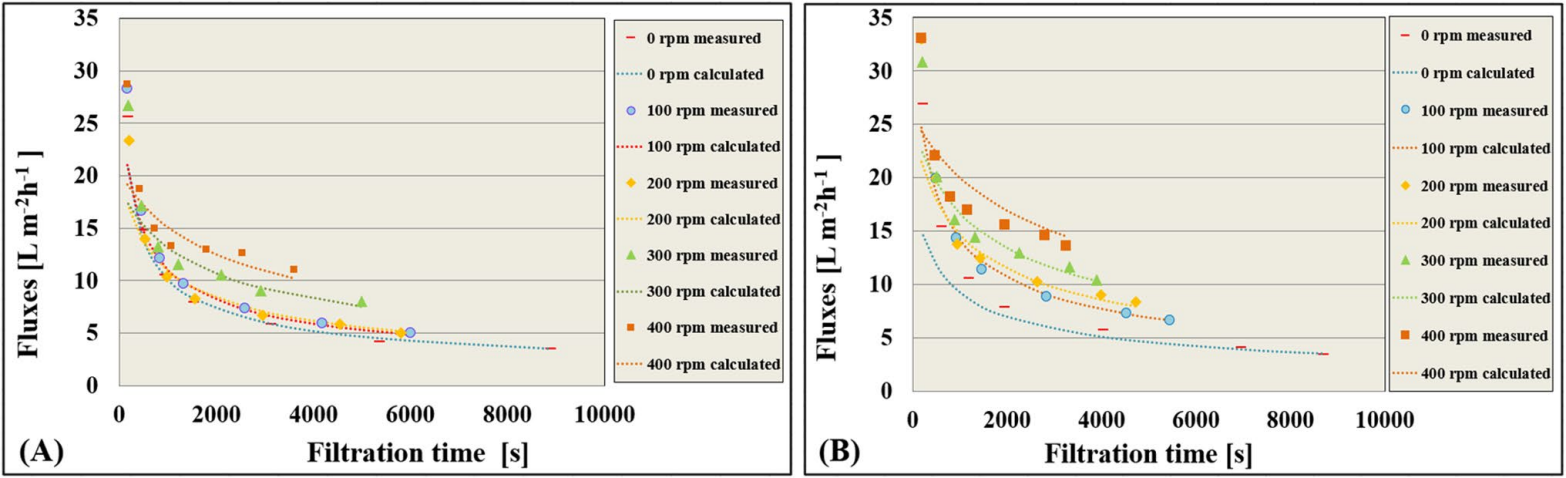
Line representation required for the accuracy of cake layer formation (Hermia) model fitting in measurements **A** without turbulence promoter and **B** with turbulence promoter

**Fig. 4:**
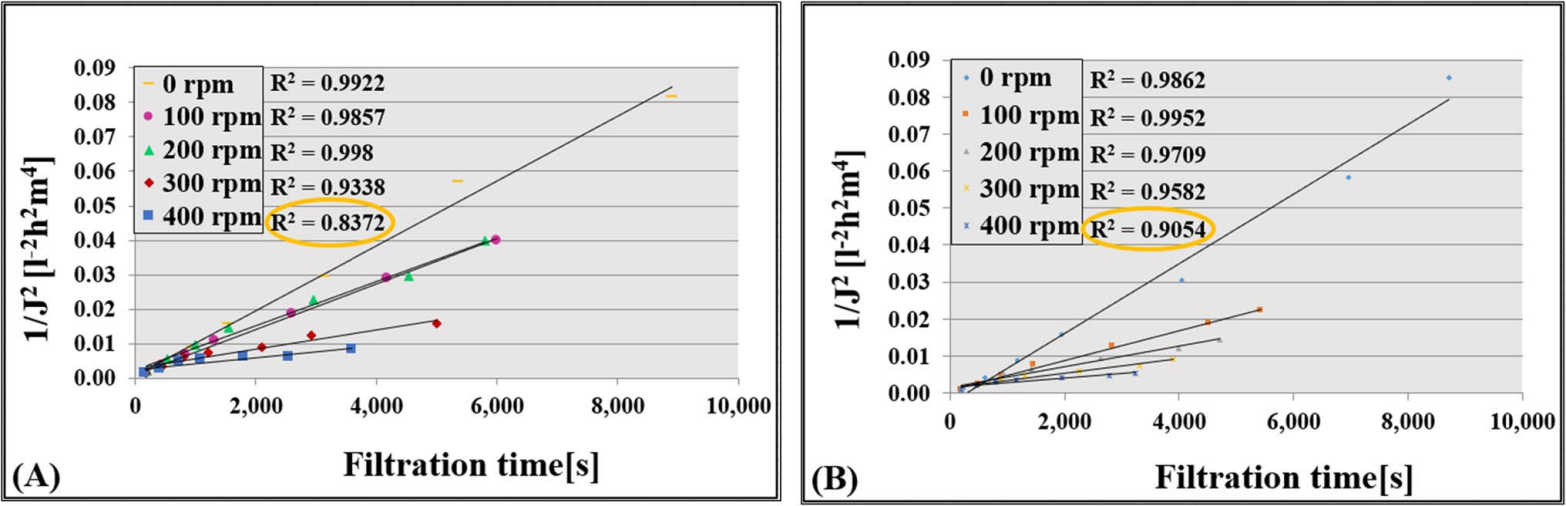
The *R*^2^ values of the cake layer formation (Hermia) model **A** without turbulence promoter and **B** with turbulence promoter

By fitting the experimental results to the four Hermia models, the cake layer formation model was found to be the most prevalent, as evidenced by the close agreement between the measured and calculated results shown in Fig. 3. The influence of the stirring speed on the filtration time was also investigated by plotting the reciprocal of the square of the flux (1/*J*^2^) as a function of time in Fig. 4. The increase in stirring speed was found to lead to a decrease in filtration time, which is consistent with the results in Fig. 3. To assess the precision of the fitting, the *R*^2^ values were calculated and presented in Fig. 4. The fitting accuracy was found to decrease with increasing stirring speed, which can be attributed to the higher tendency of the polarization layer to be destroyed at higher speeds. In general, these findings demonstrate the effectiveness of the Hermia model in predicting the filtration performance under different operating conditions.

The cake layer formation model was selected as the most appropriate model to evaluate permeate fluxes in the filtration process because of its simplicity and ability to describe fouling mechanisms effectively. It assumes the formation of a cake layer on the membrane surface, composed of accumulated particles and impurities from the feed solution, leading to decreasing permeate fluxes over time (Corbatón-Báguena et al. 2015). This model aligns well with the characteristics of dairy wastewater and the specific properties of the membrane. On the other hand, other models in the Hermia model, such as the complete blocking model, the intermediate blocking model, and the standard blocking model, have different assumptions and equations that are less suitable. The compatibility of the cake layer formation model with dairy wastewater and membrane properties makes it the most suitable choice for evaluating fouling mechanisms and permeate fluxes in this particular study.

Using the cake layer formation model, the fouling model constants (*k*_g_ [s∙m^−2^]) were also determined and compared in Table 1. On the basis of the results, it can be noticed that the *k*_g_ values decrease with increasing mixing velocities, which can be explained by the fact that the particles near the membrane surface are less able to deposit and thus form a polarization layer on the surface of the membrane.

**Table 1:** Fouling model constants (*k*_g_)

Stirring speed	Without 3DP turbulence promoter	With 3DP turbulence promoter
	*k*_g_ [s m^−2^]	*k*_g_ [s m^−2^]
Control (0 rpm)	9.43 · 10^−6^	9.42 · 10^−6^
100 rpm	6.56 · 10^−6^	4.00 · 10^−6^
200 rpm	6.39 · 10^−6^	2.76 · 10^−6^
300 rpm	2.82 · 10^−6^	1.93 · 10^−6^
400 rpm	1.72 · 10^−6^	1.25 · 10^−6^

### Retention of organic matter by membrane

**Fig. 5:**
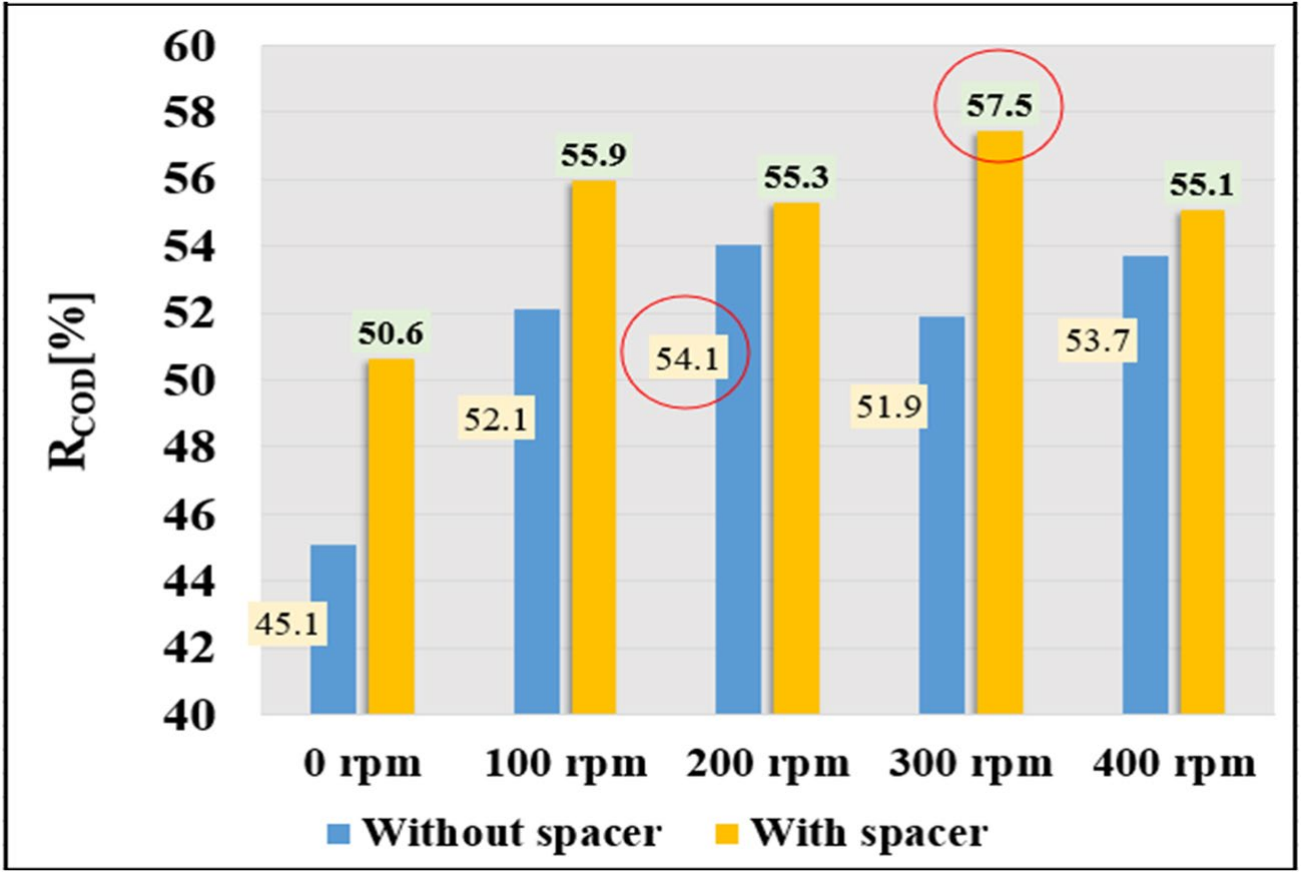
Results of the membrane retention value for organic matter

The rejection or retention values of the organic matter content by the membrane at various mixing velocities are presented in Fig. 5. Retention values ranged from 45.1 to 57.5%. The percentage of rejection increased with an increase in the stirring speed, with the highest value of 54.1% obtained at a stirring speed of 200 rpm without the 3DP turbulence promoter and 57.5% at 300 rpm with the 3DP turbulence promoter. The lowest value was recorded at 0 rpm for both cases, with and without the 3DP turbulence promoter. The use of a 3DP turbulence promoter at a stirring speed of 100 rpm produced satisfactory results. However, no significant differences were observed at 400 rpm in both cases, 53.7% and 55.1%, respectively. Integration of the 3DP turbulence promoter in all cases demonstrated improvement and process enhancement. Therefore, the differences were a 3.8% increase for 100 rpm, a 1.2% increase at 200 rpm, a 5.5% improvement at 300 rpm, and about a 1.4% improvement at 400 rpm. The results suggest that the use of the 3DP turbulence promoter does not significantly affect the retention values, but results in a slight improvement. According to Ghosal et al. (2022) multiple 3D printing methods, employing a variety of materials, are utilized to achieve customizable properties such as surface area, thickness, and roughness. The primary aim of employing these techniques is to improve the efficiency of removal of various organic pollutants during wastewater treatment.

### Membrane resistance values

**Fig. 6:**
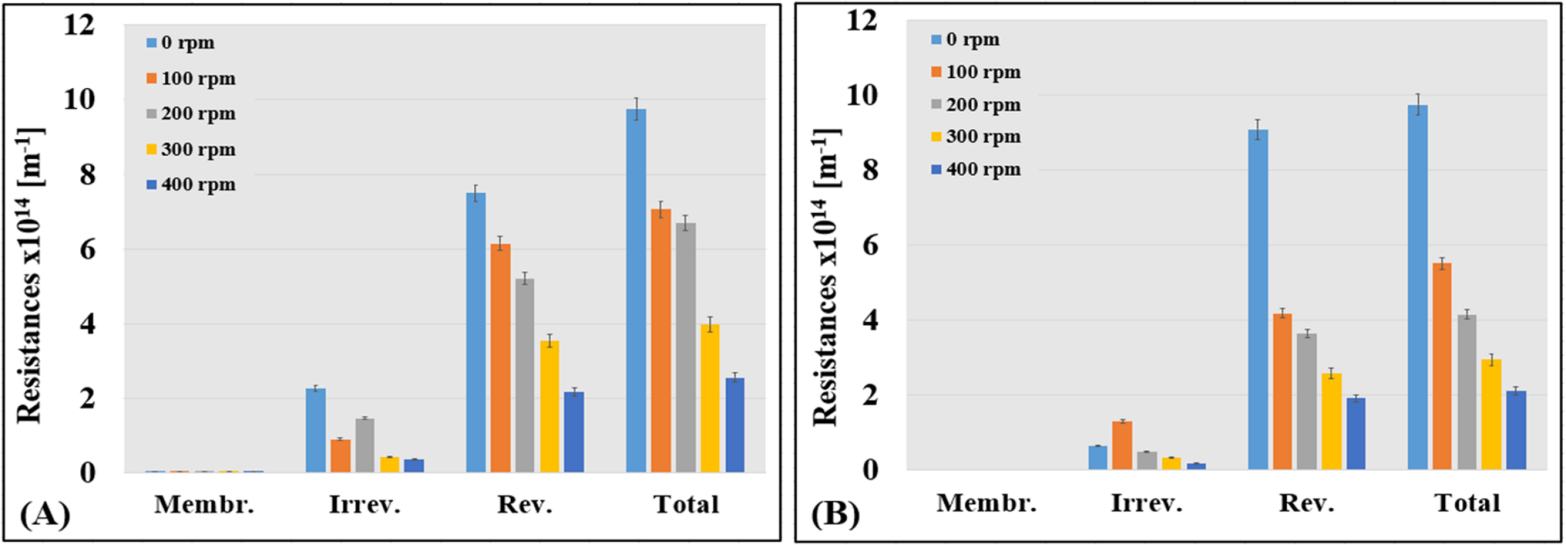
Changes in membrane resistance values due to different stirring speeds and the influence of the turbulence promoter. Resistance values for the measurements **A** without the 3DP turbulence promoter and **B** with the 3DP turbulence promoter

The hydrodynamic resistance values of the membrane, including reversible, irreversible, and total resistance, were determined using Eqs. (3), (4), (5), (6), and are presented in Fig. 6. The results indicate that the reversible resistance is the most significant, suggesting that membrane fouling occurred likely via the cake filtration model, which is a positive finding as this type of fouling is easier to remove than the irreversible one.

The impact of the stirring speed and the 3DP turbulence promoter on resistance values was investigated. Ultrafiltration without the 3DP turbulence promoter showed the highest total resistance (*R*_T_) value at a stirring speed of 0 rpm, which decreased significantly as the speed increased. Stirring speeds of 100, 200, 300, and 400 rpm reduced the *R*_T_ values by 28%, 32%, 59%, and 74%, respectively. In contrast, when using the 3DP turbulence promoter, even more significant decreases in total resistance were observed. The *R*_T_ values decreased by 43%, 57%, 70%, and 78% at stirring speeds of 100, 200, 300, and 400 rpm, respectively. This indicates that integrating the 3DP turbulence promoter into the cell improves the membrane surface shear rate, leading to more favorable flux values. Similar results were reported by Ferreira et al. (2020), who achieved a 78% increase in permeate flux using a 3DP turbulence promoter. Overall, our findings demonstrate that the use of a 3DP turbulence promoter can effectively reduce total resistance and improve filtration performance.

### Energy consumption

**Fig. 7:**
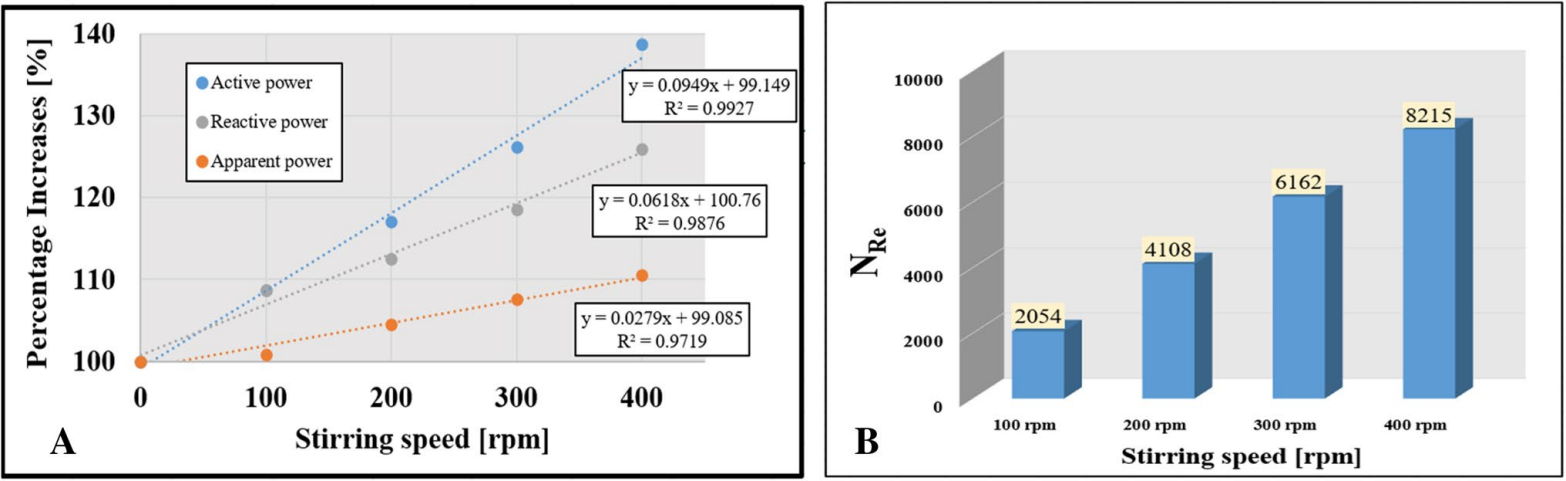
**A** Changes in the active, reactive, and apparent power values of the ultrafiltration device, and **B** Reynolds number values as a result of increasing the stirring speed without 3DP promoter integration

Through the analysis of energy consumption in the steerable membrane separation device, significant key performance indicators were identified and analyzed. Since there was no significant difference with the use of the 3DP turbulence promoter, the findings, illustrated in Fig. 7(A), demonstrate a linear relationship between the stirring speed and the active, reactive, and apparent power values for ultrafiltration without the integration of the 3DP turbulence promoter. The increase in stirring speed resulted in an increase in energy consumption, but led to lower specific energy consumption, attributed to the increase in filtration time resulting from the insertion of 3DP turbulence promoters and the subsequent increase in speed. In particular, the filtration time was observed to increase by a factor of 2.7. Furthermore, Fig. 7(B) presents a turbulent trend attributed to the Reynolds number values as a result of the increase in the stirring speed.

**Fig. 8:**
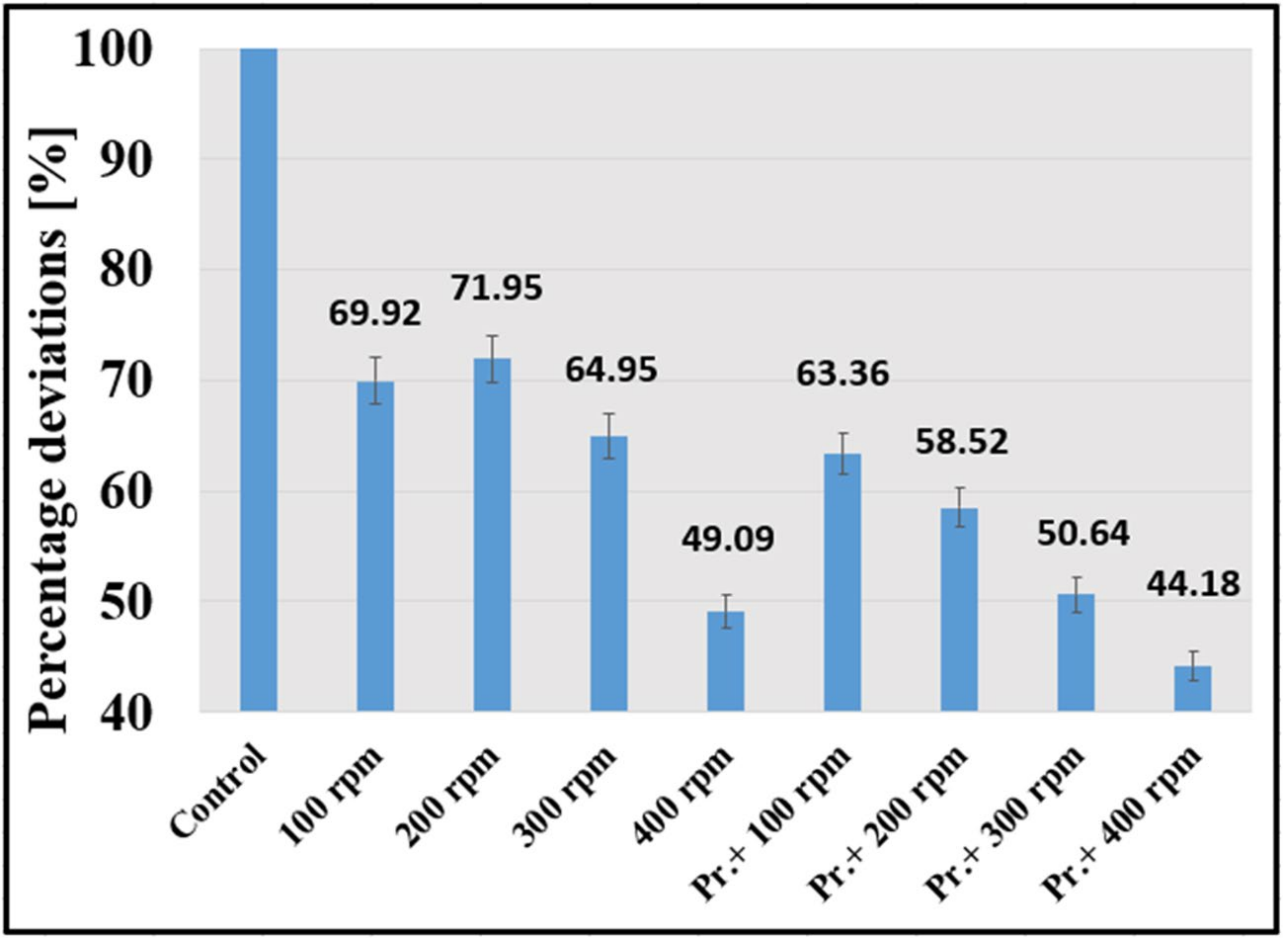
Percentage reduction of specific power values as a result of the 3DP turbulence promoter

The presentation of the measurement results through a comparison of specific energy consumption values with and without a 3DP turbulence promoter during filtration is a suitable method. As shown in Fig. 8, the specific energy consumption value for the stirring speeds (100, 200, 300, 400) with a 3DP turbulence promoter demonstrated a notable reduction in specific energy by approximately 36%, 41%, 49%, and 56% compared to the control condition.

## Limitations and challenges

The study carried out at a lab scale on the integrated 3DP turbulence promoter reveals potential challenges in scaling to a larger industrial size. For successful implementation, careful consideration of design optimization, material selection, and cost-effectiveness is necessary. The long-term stability and durability of the 3DP turbulence promoter should be assessed to ensure continuous performance in real-world industrial settings, including investigations into wear and tear, fouling, and maintenance.

Although the study shows promising results in reducing membrane fouling, more research is needed to address the various foulants of dairy wastewater in real world. Additionally, the compatibility of the turbulence promoter with various membrane types should be tested, considering that its effectiveness may vary. Evaluating the economic feasibility of implementing 3DP turbulence promoters is essential, comparing the costs with the benefits of reduced fouling and energy consumption. The environmental impacts of using 3D printing materials such as PLA in the turbulence promoter should be considered relative to alternative solutions.

The effectiveness of the 3DP turbulence promoter and the ultrafiltration system can vary based on the variability in the composition of the wastewater of different dairy products and processing methods. Regulatory compliance is crucial to meet specific regulations and standards when implementing new technologies in industrial wastewater treatment. To understand its competitiveness and advantages, a comparison with other conventional and emerging treatment methods should be performed. In conclusion, while the study presents a promising approach to reducing membrane fouling in dairy wastewater treatment, more research and development is required to address the limitations and challenges mentioned before implementing it on a larger scale in real-world industrial applications.

## Conclusions

This study revealed that the integration of the 3DP turbulence promoter precipitated a reduction in the duration of filtration and a substantial 2.7-fold increase in the permeate flux relative to the control setup. The analysis revealed the suitability of the cake layer model, among the Hermia models, for assessing permeate fluxes. Examination of 1/*J*^2^ values signified that the use of the 3DP turbulence promoter yielded analogous linear trends in mean flow and relative permeate fluxes. The investigation demonstrated a 5.5% increase in the rejection of organic matter when using the 3DP turbulence promoter at 300 rpm. On the contrary, the *R*_T_ values exhibited a 78% decrease at a stirring speed of 400 rpm. Meanwhile, within the series resistance model, overall membrane resistance with the integrated 3DP turbulence promoter witnessed a decline. Evaluation of the ratio of total resistance values elucidated that the reversible resistance values were the highest, implying that fouling predominantly followed the cake layer model, consistent with the Hermia model outcomes. Furthermore, the examination of energy consumption revealed an increase in practical energy values with increasing speed, while specific power values decreased by 44.18% at 400 rpm due to the reduced filtration time facilitated by the promoter and the escalation in stirring speed.

## Data Availability

Data will be made available on request.
